# SDG partnerships may perpetuate the global North–South divide

**DOI:** 10.1038/s41598-021-01534-6

**Published:** 2021-11-25

**Authors:** Malgorzata Blicharska, Claudia Teutschbein, Richard J. Smithers

**Affiliations:** 1 Natural Resources and Sustainable Development, Department of Earth Sciences, Uppsala University, Uppsala, Sweden; 2 Air, Water and Landscape Sciences, Department of Earth Sciences, Uppsala University, Uppsala, Sweden; 3 Ricardo Energy & Environment, Gemini Building, Fermi Avenue, Harwell, Didcot, OX11 0QR UK

**Keywords:** Environmental social sciences, Sustainability

## Abstract

The 2030 Agenda for Sustainable Development gives equal emphasis to developed (“Northern”) countries and developing (“Southern”) countries. Thus, implementation of the Sustainable Development Goals (SDGs) demands coherent collaboration to transform society across all countries. Yet, there has been little research published on SDG partnerships and this is the first study to explore the extent to which partners from Northern and Southern countries are involved in them and their focus. It identifies that involvement is unequally distributed and may perpetuate the North–South divide in countries’ resources, including access to data and scientific capacities. Most notably, partners from low-income countries are involved in far fewer partnerships than partners from countries in all other World Bank income categories, although the former are least able to develop sustainably. As such, all those promoting sustainable development from governmental, private and third-sector organisations need to address global inequalities in establishing and implementing SDG partnerships if, collectively, they are to facilitate delivery of Agenda 2030.

## Introduction

The 2030 Agenda for Sustainable Development was adopted in 2015 by all United Nations (UN) Member States. Its 17 Sustainable Development Goals (SDGs) and 169 targets build upon the Millennium Development Goals (MDGs). While the MDGs focused on actions in developing (“Southern”) countries, the SDGs give equal emphasis to implementation in developed (“Northern”) countries^1^. However, there is a global North–South divide in countries’ resources, including access to data and scientific capacities. For example, low-income countries have much less investment in research and development and far fewer researchers, when compared to higher income countries^2,3^. This has substantial implications for the development and implementation of policies and practices. The negative consequences of the divide for how science is designed, produced and communicated and how action on the ground is implemented have been highlighted at all scales from international to local^4,5^ and across various themes and sectors^6–9^. Specifically, it leads to policy development and implementation shaped by a Northern agenda rather than the needs and priorities of Southern countries^5,8^, preventing global sustainable development and, thereby, fulfilment of Agenda 2030. Thus, the SDGs present numerous challenges for transforming society through coherent collaborative efforts involving all countries^10^. While each country is responsible for its own sustainable development^11^, global telecoupling^12^ means that many SDG actions should be implemented by countries in partnership.

SDG 17 focuses specifically on strengthening implementation of the SDGs, including through North–South, South-South and triangular cooperation, and multi-stakeholder partnerships^13^,​ which can be public and private^10^. Over recent years, the importance of multi-stakeholder partnerships has been increasingly recognized by UN Member States and leading international development institutions^14^. SDG Target 17.16 highlights that multi-stakeholder partnerships can “mobilize and share knowledge, expertise, technology and financial resources, to support the achievement of the SDGs in all countries, in particular developing countries”^15^. While partnerships may not be a panacea for global inequalities, they offer opportunities for people and institutions from different contexts to negotiate social relations and power that can lead to more respectful, equal implementation^16^. It has been specifically suggested that Northern countries should “co-produce knowledge, technologies, and processes for sustainability”^17^ with Southern countries to support development of their capacities^18,19^. The equitable involvement of southern partners in such co-production is important to ensure that resultant knowledge systems are credible, salient and legitimate^20^.

Despite the acknowledged importance of multi-stakeholder partnerships for sustainable development, there has been limited research on their focus, which countries are involved, and relationships between them. Additionally, while SDG 17 highlights the need to enhance Southern countries’ capacity to implement the SDGs (Target 17.16), little is known about its achievement. While there are some reports on SDG partnerships^21–23^, very few have focused on how they address the global North–South divide^24^. Thus, our paper aims to explore the extent to which partners from Northern and Southern countries are involved in SDG partnerships globally, in different regions of the world, and the SDGs on which they focus. We use World Bank income categories^25^ in applying the descriptors “North” or “Northern” to high-income countries, and “South” or “Southern” to countries in all other income categories^25^. Our analyses use data collected from the UN’s SDG Partnerships Platform.

## Results

### Countries in the partnerships

A total of 4521 partnerships were registered on the UN’s SDG Partnerships Platform in July 2019. Of those, 1645 partnerships (36%) referred to an organisation (e.g. the UN or the European Commission) or a broad geographical focus (i.e. without identifying partners from a specific country), with most referring to “oceans” (863; 19%) or “global” (371; 8%). Subsequently, our analyses of “countries’ involvement” addressed the 2876 partnerships (64%) that specifically identified partners from one or more countries. Of these partnerships, 1724 (60%) involved partners from at least one Southern country and 1309 (45%) included partners from one or more Northern countries.

**Figure 1:**
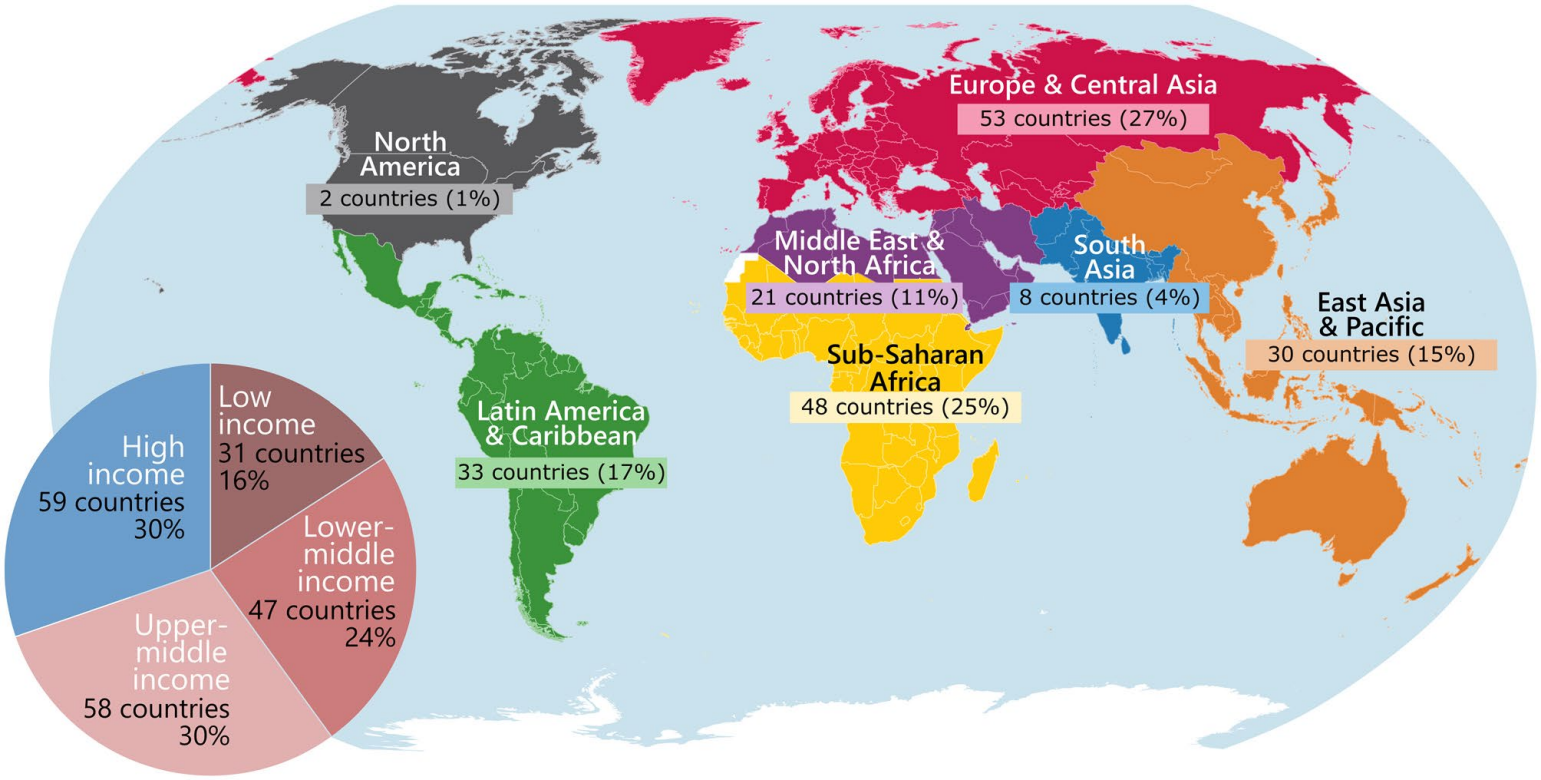
Distribution of partners’ countries by World Bank income category and region. The number and percentage of partners’ countries involved in the 2876 partnerships that specifically identified partners from one or more countries are shown by World Bank income category (pie-chart) and by World Bank region (large map). Total number of partners’ countries: 195. Map generated in QGIS 3.12 (https://qgis.org/) and further modified in Inkscape 0.92.5 (https://inkscape.org/).

Partners from a total of 195 countries were involved in at least one registered partnership (Fig. 1). Of those, 30% (59) were from high-income Northern countries and the remainder (136) were from Southern countries: upper-middle-income (30%), lower-middle-income (24%) and low-income (16%) (Fig. 1, pie chart).

The partners’ countries were unevenly distributed across the World Bank’s seven world regions (Fig. 1), with the greatest number located in Europe & Central Asia (53; 27%), and Sub-Saharan Africa (48; 25%). The number of partnerships in which partners from these countries were involved ranged from 1 to 231, with an average of 29 partnerships per country. Partners from 174 (90%) of the countries were involved in less than 60 partnerships, while partners from 93 (48%) of the countries were involved in less than 20 partnerships.

**Figure 2:**
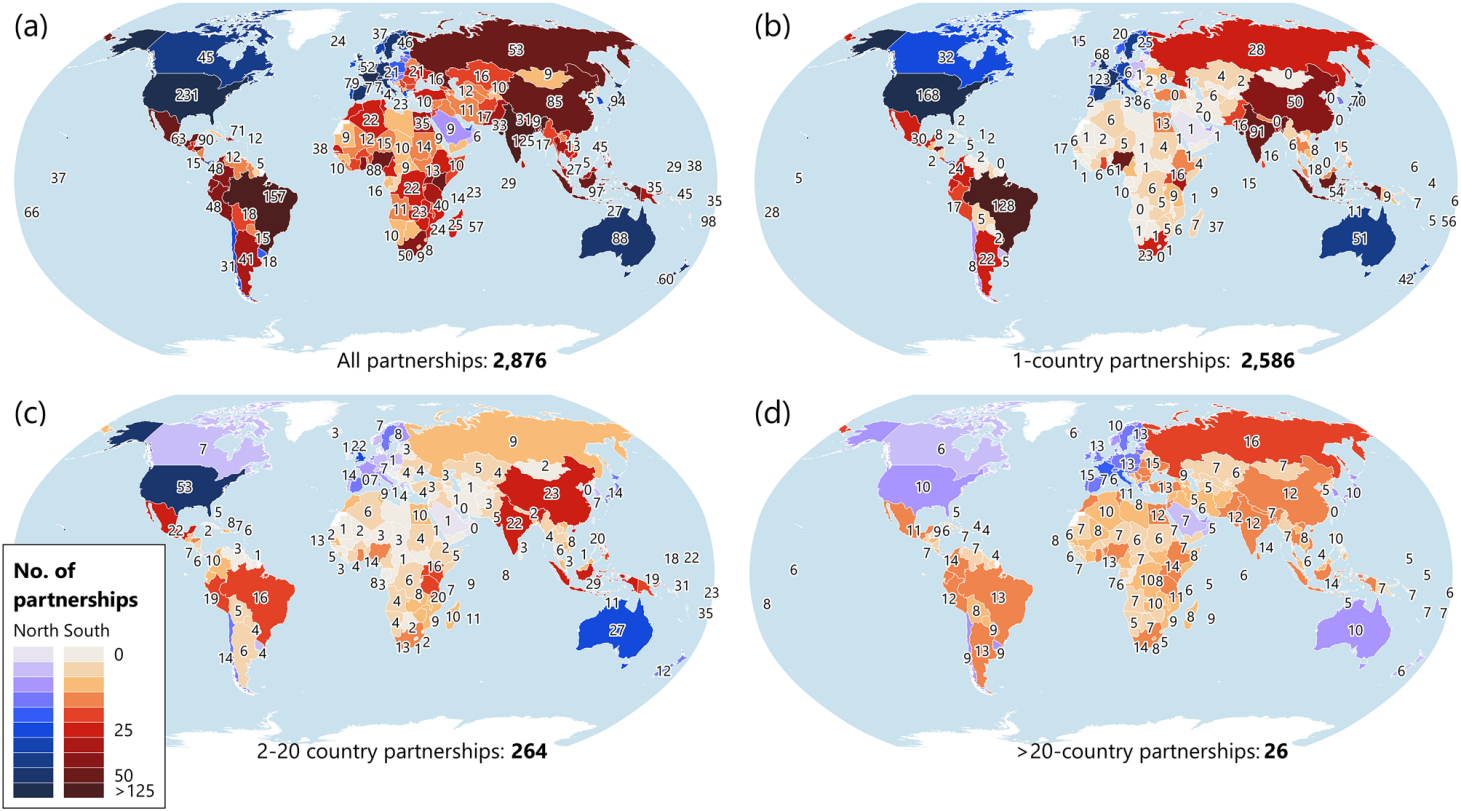
Distribution of the number of partnerships that specifically identified partners’ countries. Overview of the number of partnerships that specifically identified partners from: (**a**) one or more countries; (**b**) only one country, i.e. domestic partnerships; (**c**) 2 to 20 countries; and (**d**) more than 20 countries. Northern countries (high income) are shown in shades of blue; Southern countries (upper-middle, lower-middle and low income) are shown in shades of red. Maps generated in QGIS 3.12 (https://qgis.org/). Larger versions of the components of this figure are included in Supplementary Material.

Registered partnerships that specifically identified partners from one or more countries (Fig. 2a) included 2586 (90%) partnerships with partners from only one country, i.e. domestic partnerships (Fig. 2b), 264 (9%) partnerships with partners from 2 to 20 countries (Fig. 2c), and 26 (1%) partnerships with partners from more than 20 countries (Fig. 2d). On average, each country had 13 domestic partnerships, 7 partnerships involving partners from 2 to 20 countries, and 13 partnerships with partners from more than 20 countries.

**Figure 3:**
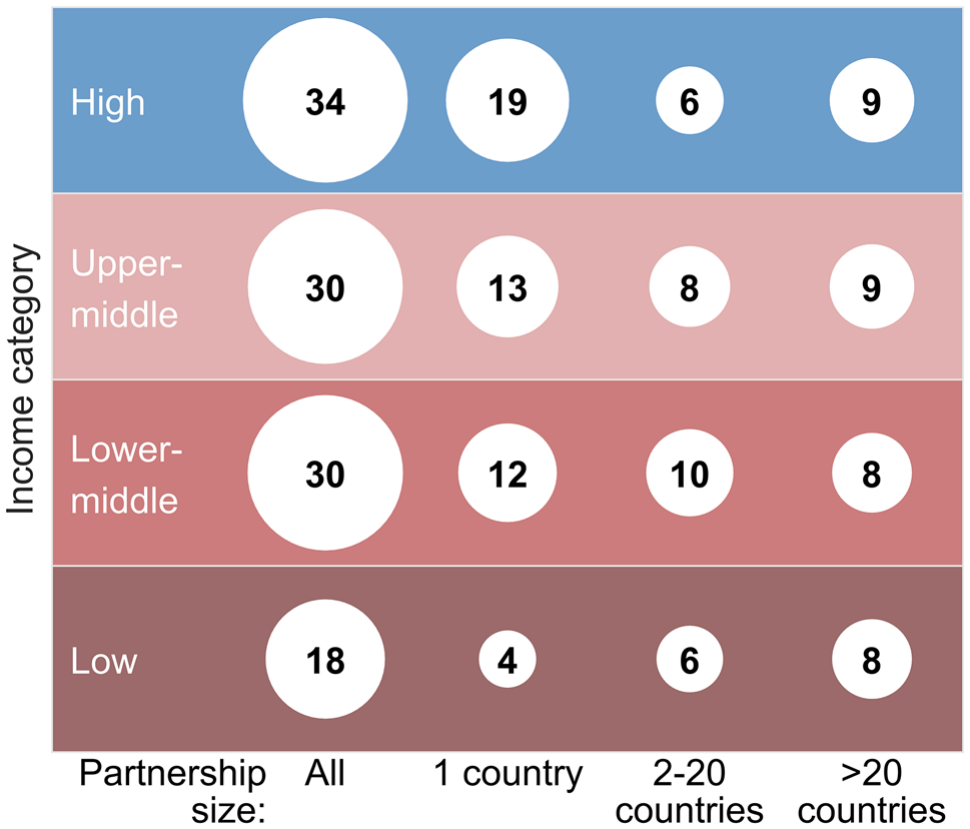
Average number of partnerships per partners’ countries by partnership size and country income category. Figures in the first column are not the sum of figures in the subsequent columns, as all are average figures.

There were some clear differences in the average number of partnerships in relation to the income category of partners’ countries (Fig. 3). Across all sizes of partnerships, partners from high-income (Northern) countries participated in almost twice as many partnerships (34 per country) as those from low-income countries (18 per country). Partners from low-income countries were also involved in considerably fewer domestic partnerships (4), as compared with those in other income categories (12 to 19), on average. However, partners from countries across all income categories were, on average, party to a similar number of partnerships with partners from 2 to 20 or more than 20 countries (6 to 10 and 8 to 9 partnerships, respectively).

### Partnerships’ focal SDGs

**Figure 4:**
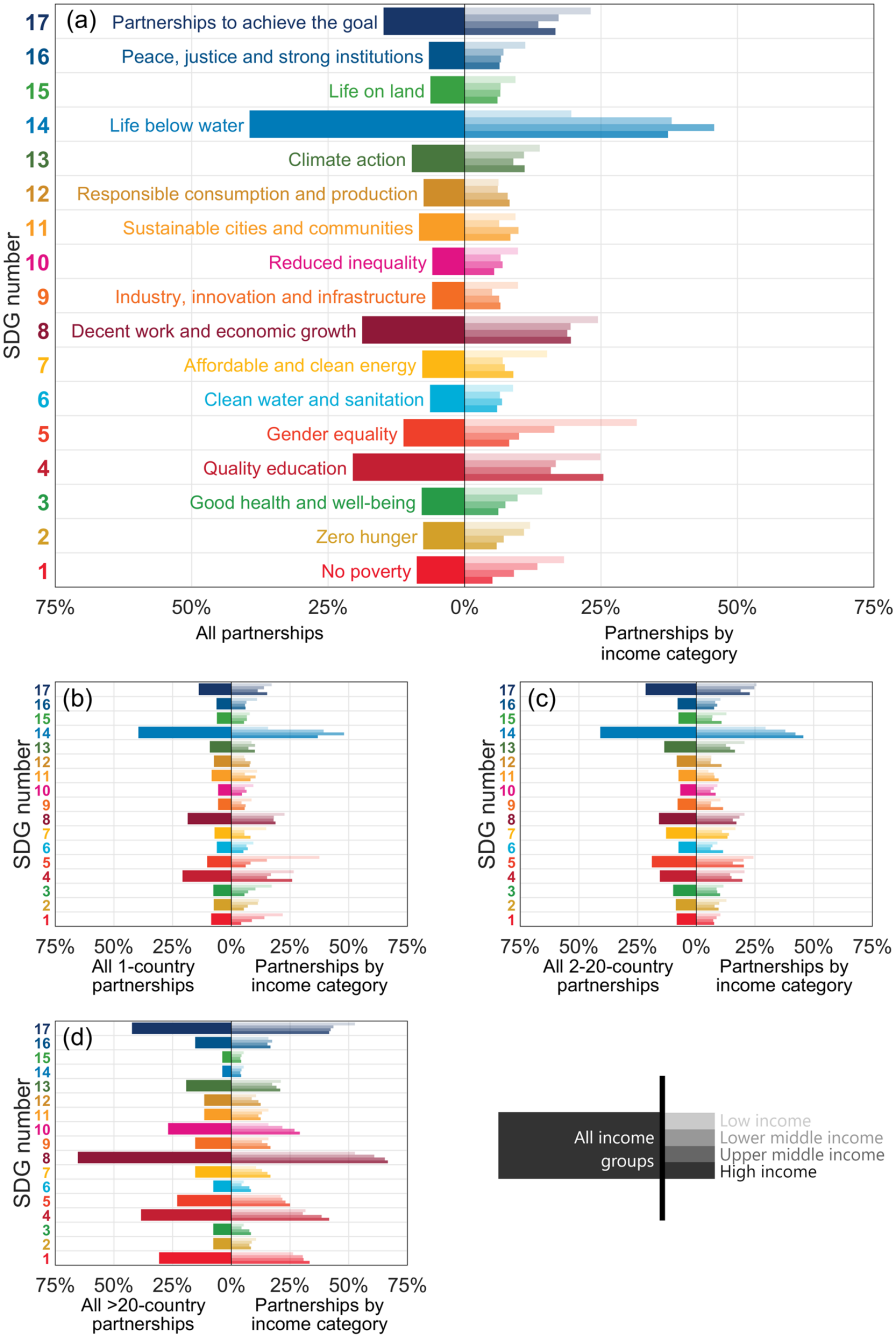
Partnerships’ focal SDGs. The SDGs addressed by (**a**) all partnerships that specifically identified partners from at least one country, (**b**) domestic partnerships, (**c**) partnerships involving 2 to 20 countries, and (**d**) partnerships with more than 20 countries. The bars on the left of Figs. 4a to 4d represent the percentage of partnerships that address each SDG. The bars on the right of Figs. 4a to 4d represent the percentage of partnerships that address each SDG involving partners from countries in each income category (represented by different intensities of colour). Note that many partnerships include partners from countries across multiple income categories and address more than one SDG. Larger versions of the components of this figure are included in Supplementary Material.

Based on the information included in the UN database, we identified each partnership’s focal SDGs. In general, all partnerships that identified partners from at least one country (Fig. 4a) focused most strongly on SDG 14 (Life below water) and then in descending order on SDGs 4 (Quality education), 8 (Decent work and economic growth), 17 (Partnerships for the goals) and 5 (Gender equality). This was also true more specifically of domestic partnerships (Fig. 4b). However, while partnerships that identified partners from 2 to 20 countries also focused most strongly on SDG 14, they then focused in descending order on SDGs 17, 5, 4, and 8 (Fig. 4c). There was an increasing focus on SDG 17 with size of partnership. The focus of the largest partnerships was, in general, quite different from partnerships that identified partners from 20 or fewer countries (Fig. 4b,c), as they focused most strongly on SDG 8 and then in descending order on SDGs 17, 4, 1 (No poverty), and 10 (Reduced inequalities). In addition, few of the largest partnerships focused on SDG 14 (Fig. 4d).

There were notable differences in the relative proportion of partners from countries in different income categories involved in partnerships focused on some SDGs. In general, across all partnerships that identified partners from at least one country (Fig. 4a) and, specifically, for domestic partnerships (Fig. 4b), the percentage of partnerships focused on SDGs 1, 2 (Zero hunger), 3 (Good health and well-being), and 5 decreased with increasing income category of the countries involved. In contrast, for SDG 14 the reverse was broadly true, except for high-income countries, and for SDG 4 a higher proportion of low-income and high-income countries was involved than middle-income countries. Notably, the relative proportion of partners from countries in different income categories involved in partnerships that focused on SDG 13 (Climate action) was broadly similar within each size category of partnerships and increased across all income categories in the same way with increasing size of partnership.

### Country-wise relations between partners

**Figure 5:**
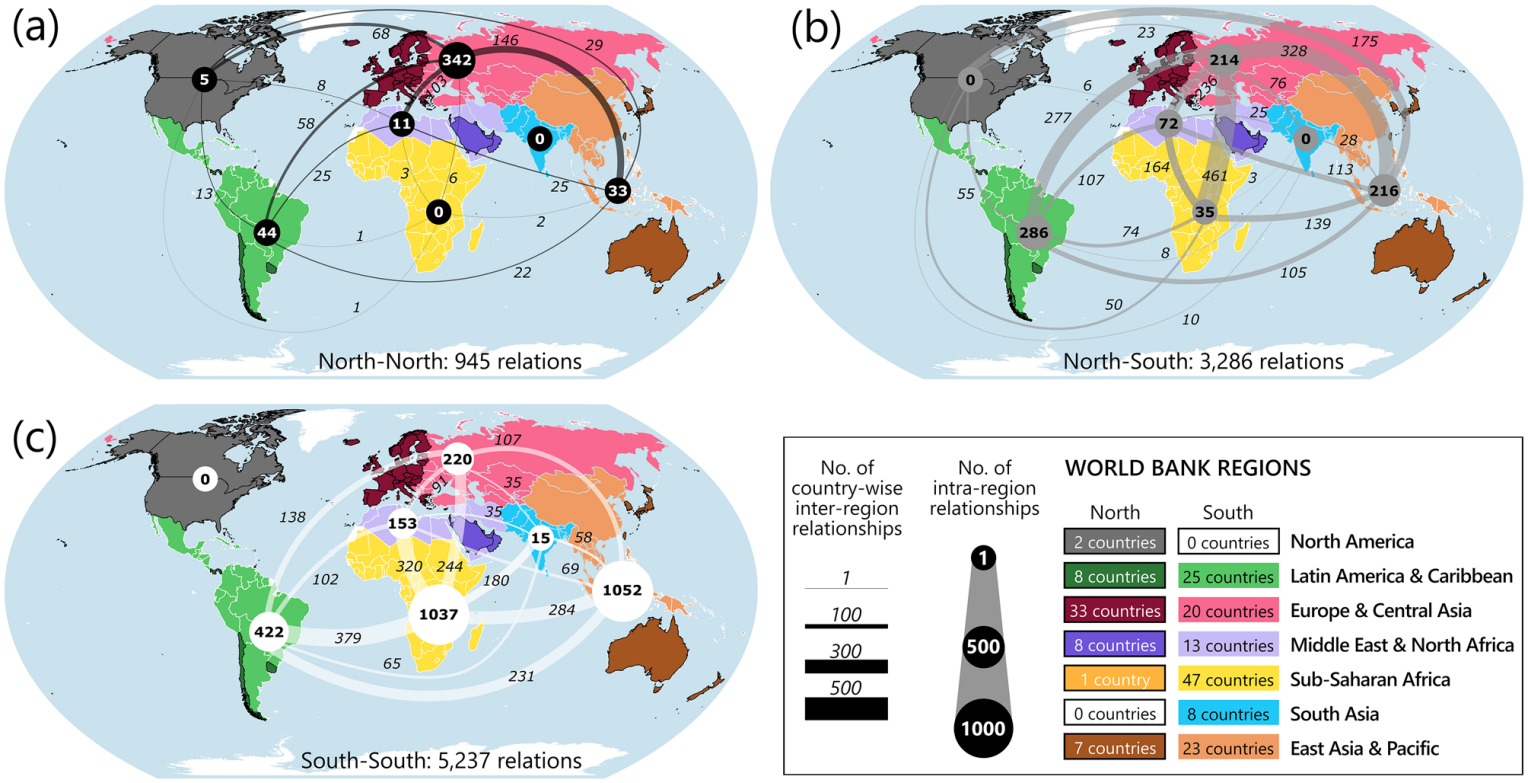
Number of country-wise relationships within and between World Bank regions. The types of relationships are indicated by different coloured circles (within regions) and lines (between regions): (**a**) North–North relationships (black), (**b**) North–South relationships (grey) and (**c**) South-South relationships (white). The size of circles and the figures in the circles represent the number of relationships between partners from different countries within each region. Line thickness and the figures in italics associated with the lines represent the number of relationships between partners from different regions. Numbers in the coloured boxes of the legend identify the respective numbers of Northern countries and Southern countries in each region included in the analysis. Maps generated in Arcgis Desktop 10.7 (https://www.esri.com/en-us/arcgis/products/arcgis-desktop/overview) and further modified in Inkscape 0.92.5 (https://inkscape.org/). Larger versions of the components of this figure are included in Supplementary Material.

In order to analyse the country-wise relationships between partners from different countries and World Bank regions, we assumed that a partner from a country had relations with partners from all other countries within the same partnership. The analysis focused only on those partnerships that involved partners from 2 to 20 countries (see “Methods“ for details), i.e. 264 partnerships. These partnerships involved 9468 such country-wise relationships. Approximately 10% (945) of the relationships solely involved partners from Northern countries, i.e. North–North (Fig. 5a), 35% (3286) were between partners from Northern and Southern countries, i.e. North–South (Fig. 5b), while most (55%; 5236) solely involved partners from Southern countries, i.e. South-South (Fig. 5c). Approximately 36% (342) of all North–North relationships were between partners within Europe & Central Asia region, and a further 15% (146) were between partners from Europe & Central Asia and East Asia & Pacific. Most North–South relationships (2463; 75%;) were between partners in different regions, most commonly between partners in Europe & Central Asia and East Asia & Pacific (328; 10%), and between partners in Europe & Central Asia and Latin America & Caribbean (277; 8%). A large proportion of South-South relationships (55%; 2898) were between partners from countries within the same region, particularly within East Asia & Pacific (1052; 58%) and Sub-Saharan Africa (1037; 42%). The remainder of South-South relationships (45%; 2338), between partners in different regions, mostly involved partners from Sub-Saharan Africa (1407; 60%).

**Figure 6:**
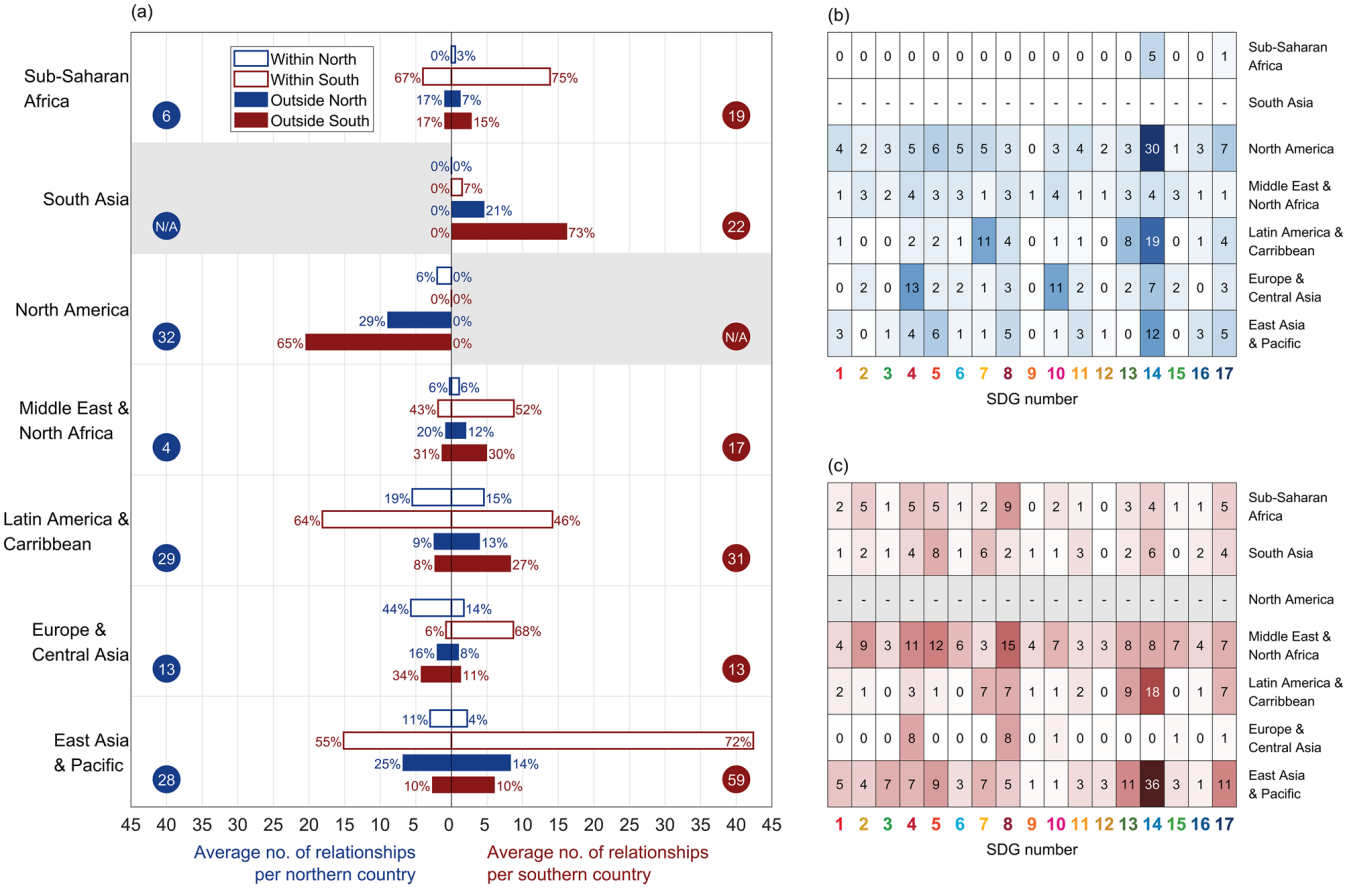
Comparison of the average number of country-wise relationships. For: (**a**) Northern countries or Southern countries in different World Bank regions (blue or red circles), separated into relationships between partners in different Northern or Southern countries (blue or red bars), within or outside each region (transparent or filled bars), where the number at the end of each bar is the percentage of that type of relationship within that region; (**b**) Northern countries in different World Bank regions by SDG; and (**c**) Southern countries in different World Bank regions by SDG. Larger versions of the components of this figure are included in Supplementary Material.

The average number of country-wise relationships differed among World Bank regions (Fig. 6a, circles). For Northern countries, overall those in North America had the greatest number of relationships per country (32), closely followed by those in Latin America & the Caribbean (29) and East Asia & Pacific (28), while for Southern countries, those in East Asia & Pacific had the greatest number of relationships per country (59 relationships).

Not only did the average number of country-wise relationships differ among World Bank regions, so did the frequency of different types of relationships in which partners were involved, i.e. with partners in Northern or Southern countries, within or outside their own region (Fig. 6a). Relationships between Northern partners were most prevalent in North America and in East Asia & Pacific. In both cases, the greatest proportion of such relationships, 29% (18 out of 63) and 25% (48 out of 194) respectively, were with partners from Northern countries in other regions. In contrast, partners from Northern countries, in general, had most relationships with partners from Southern countries within the same region, e.g. in East Asia & Pacific (106 out of 184; 55%), Latin America & Caribbean (145 out of 228; 64%), and Sub-Saharan Africa (4 out of 6; 67%). Two regions were exceptions: Europe & Central Asia, where many relationships (188 out of 423; 44%) were with other Northern partners within the region; and North America, where most relationships (41 out of 63; 65%) were with Southern countries outside the region. Partners from Southern countries, in general, had most relationships with partners from other Southern countries within the same region, with most such relationships in East Asia & Pacific (975 out of 1358; 72%), Europe & Central Asia (174 out of 257; 68%), and Sub-Saharan Africa (651 out of 871; 75%). The only exception was South Asia where partners had most relationships (130 out of 179; 73%) with partners in Southern countries outside the region.

The average number of country-wise relationships also differed depending on the SDGs that were addressed by partnerships in which partners from countries in a region were involved (Fig. 6b,c). The highest average number of relationships for Northern countries (Fig. 6b) was associated with partnerships focused on SDG 14, particularly those with partners from North America, followed by those involving partners from Latin America & the Caribbean, and East Asia & Pacific. There were also more relationships for Northern countries associated with partnerships focused on SDG 4 and/or 10 involving partners from Europe & Central Asia, and on SDG 7 with partners from Latin America & the Caribbean than for any other regions or focal SDGs. For Southern countries, by far the highest average number of country-wise relationships was associated with partners from East Asia & Pacific, and Latin America & the Caribbean, involved in partnerships focused on SDG 14. In addition, there were more relationships for Southern countries associated with partnerships focused on SDG 8, as well as 5 and/or 4, involving partners from the Middle East & North Africa, and on SDG 13 and/or 17 with partners from East Asia & Pacific than for any other regions or focal SDGs.

## Discussion

The analysis highlighted several issues indicating that implementation of the SDGs may suffer from unequal distribution of effort globally, potentially perpetuating the North–South divide^5,8^.

### Most partnerships recorded in the UN database were domestic partnerships

The idea of multi-stakeholder partnerships to tackle global sustainability challenges, as introduced by SDG 17, has gained traction recently. However, the actual distribution of partnerships may seemingly have reflected the ease with which they were developed rather than sustainable development needs. For example, it may have been easier to develop partnerships within a country or with partners in nearby countries because of pre-existing relations, logistics or familiar sources of funding.

### Partners from low-income countries participated in fewer partnerships, as compared to partners in all other income categories

This may suggest that partners from low-income countries did not have the capacity or resources to get involved in partnerships. In addition, it is conceivable that Northern countries favoured working with partners from middle-income categories for a range of different reasons. For example, it may be that they had more in common, had greater resources of interest, could form more strategic alliances, were already making progress, and/or were less challenging to work with than partners from low-income countries. A recent study by Bull and McNeill^26^ that analysed public–private partnerships focused on SDGs revealed similar domination by actors from Northern countries, particularly in Europe (followed by Africa and Oceania), and found correlation between GDP per capita and the number of partnerships in which partners from a country was involved. Partners from high-income countries also previously dominated public–private partnerships focused on the MDGs^27^.

### The focus of partnerships differed depending on the number of countries from which partners were involved

The largest partnerships (with partners from more than 20 countries) focused substantially more on SDG 8, and the percentage of partnerships focused on it increased with increasing income category of the countries from which partners were involved. It may be worrying that the largest and, thus, potentially most powerful partnerships focused on economic growth. This focus also reflects a recent critique that the SDGs place inappropriate emphasis on development considerations, instead of on sustainability^28^. At the same time, few largest partnerships focused on SDG 14, which may reflect that the sustainable development of our oceans is largely neglected^29^. However, the marine environment was a considerable focus for partnerships with fewer than 20 partners, perhaps where nearby countries shared interests in what is one of our greatest commons.

### The focus of partnerships also differed depending on the income category of the countries involved

Those with partners from low-income countries focused more on SDG 1, 2, 3, 5 and 7 than those involving partners from higher income countries. This is striking, as partners from high-income countries might also have been expected to focus on these goals from an altruistic, humanitarian perspective and in their countries’ own vested interests, given potential transnational climate impacts and spill-over effects, such as on regional conflict, mass migration, and spread of disease^30–32^. Indeed, both those potential motivations may perhaps be reflected in the greater involvement of partners from low-income and high-income countries, as compared with those from middle-income counties, in partnerships focused on SDG 4. In contrast, relatively few partners from low-income countries were involved in partnerships focused on SDG 14, which seems logical, as they are most likely to be landlocked^33^.

The relative focus on climate action (SDG 13) was similar for partners from countries across all income categories. However, our results do not disaggregate whether the focus on SDG 13 was primarily on climate mitigation and/or adaptation. It might have been anticipated that partnerships would have involved more partners from Northern countries when focused on climate mitigation, and from Southern countries when focused on climate adaptation, reflecting that G20 countries are responsible for almost 80% of global emissions^34^, while Southern countries have suffered most from extreme weather events^35^ and are perceived as disproportionately vulnerable to climate change^36,37^.

### Partnerships with partners from more than one country included North–North country-wise relationships least frequently, relatively few North–South relationships, and mostly South–South relationships

This may perhaps be explained by a legacy of thinking from the MDGs having led partners from Northern countries to view SDGs as still a Southern rather than global and domestic concern for all and, hence, neither prioritising North–North nor North–South relations. Indeed, North–South relationships were most commonly between partners in Europe & Central Asia and East Asia & Pacific, and between partners in Europe & Central Asia and Latin America & Caribbean, which may simply reflect colonial legacies and/or common languages (e.g. English, Spanish, French, Portuguese). Furthermore, partners from Southern countries may have preferentially formed partnerships with other Southern countries with common challenges and experiences from whom they could most readily learn, or in a quest to avoid uneven power relations with Northern countries. Indeed, South-South collaboration has been highlighted as important for strengthening the capacities of Southern countries^5^. However, a large proportion of South-South relations were with partners from countries within the same region, which may suggest an inability to engage in partnerships elsewhere without Northern involvement.

### Finally, at a regional level, unequal distribution of effort globally may reflect geopolitics

For example, partners in North America had the greatest number of North–North relationships, mostly with partners from other regions, perhaps reflecting the global influence of the US. In contrast, the differing situation in Europe & Central Asia, where most North–North relationships were between partners within the region, probably reflects the EU’s governance. In general, most North–South relationships were between partners within the same region, possibly reflecting self-centred Northern concerns about nearby Southern countries as potential sources of transnational impacts or spill-over effects^30^.

## Conclusions and recommendations

Our study highlights that the implementation of SDGs, through partnerships, is not equally distributed globally and may, thus, perpetuate the existing North–South divide. Partners from low-income countries, particularly, were involved in far fewer partnerships generally and domestic partnerships specifically those in any higher income categories. Yet, low-income countries are least able to advance their sustainable development independently, most in need of capacity-building support, and most disadvantaged by the North–South divide. So, to fully address Agenda 2030, all those involved in encouraging and establishing partnerships for SDGs (e.g. international funders and development organisations, national policymakers, research institutions, the private sector, non-governmental organisations, and potential partners themselves) need to address challenges posed by existing global inequalities in the design and implementation of partnerships. In particular, the perceptions and capacity-building needs of partners from low-income countries require meaningful consideration^38^.

Investment by funders in the establishment of multi-country partnerships should be encouraged, specifically focusing on North–South partnerships and supporting partners from low-income countries. Moreover, there is a need for further research regarding the impact of the North–South divide on the focus of SDG partnerships, as our analysis identifies that the focus of partnerships differs depending on the income categories of the partners involved. As such, there is a need to explore whether the potential bias in focal SDGs addressed by partnerships may reflect the interests of higher income countries that have greater capacity to further their agenda, and how to encourage more investment by funders in low-income countries’ priorities. There is also a need for better reporting and data gathering in relation to SDG partnerships in order to track their work on SDGs globally, as well as its progress in bridging the North–South divide. The UN’s SDG Partnerships Platform that we used to gather the data for our study does not ensure data entry of parameters relevant to the North–South divide, e.g. many descriptions of SDG partnerships do not include information about the countries or the types of organisations involved.

All the above conclusions and recommendations point to Northern countries, international funders and development organisations needing to pay greater heed to SDG 17, its targets (e.g. 17.2, 17.6, 17.9 and 17.16) and associated indicators. SDG 17 will only truly strengthen implementation of the SDGs in ways that span the North–South divide if they listen and respond to low income countries’ needs rather than pursue a Northern agenda. This includes recognizing that seeking to leverage private finance may inevitably lead to an inappropriate emphasis on SDG 8 and that the primary need is to encourage sustainability rather than development.

North–South partnerships need to be forged on mutual terms in a non-discriminatory and equitable way, as stipulated by Agenda 2030. This was not the case during the implementation of the MDGs, where North–South partnerships were most commonly developed from a Northern perspective, according to Northern priorities rather than attentive of Southern needs and views^39^. However, Agenda 2030 offers an opportunity to address this shortcoming and promote more balanced implementation of global sustainable development, while bridging the North–South divide. To conclude, it is vital to “Mind the gap” between Northern and Southern countries in establishing and implementing partnerships for sustainable development or, collectively, they will fail to provide the coherent collaboration needed to transform society across all countries.

## Methods

Data about SDG partnerships were collected from the UN’s SDG Partnerships Platform (https://sustainabledevelopment.un.org/partnership/browse), which maintains the UN’s global online register of voluntary commitments and multi-stakeholder partnerships established in support of the 17 SDGs. It is the most comprehensive global database of partnerships implementing the SDGs, although we acknowledge that it limits our analyses in a few ways. Firstly, the database is voluntary, so the registered partnerships will not represent all those that exist globally. However, registering a partnership on the database reflects a broad commitment to SDG implementation and, as such, provides a worthwhile overview of important actors working towards Agenda 2030. Secondly, while the Platform provides a standard template for partnership information, there is variation in the data provided. For example, some partnerships do not provide clear information about the partners’ countries, so could not be included in our analyses. In addition, the information in the database may list partners’ countries but does not define partners’ roles, i.e. whether those countries listed are the ones from which partners were involved or the ones where the partnerships were active. Thus, our study only uses the names of partners’ countries and does not analyse their roles. Finally, the database does not enable checking of whether partnerships are actively implementing the SDGs or are just partnerships “on paper”. The status “not active” in the UN’s register only means that a partnership has not submitted a voluntary progress report, so it may be still active. Thus, our study analyses partnerships registered on the UN Platform, no matter their activity status.

We collected and saved information in an Excel database on each partnership: name, code, brief description, the focal SDGs addressed, and partners’ countries. Data on a total of 4521 partnerships were gathered by 18th August 2019, involving partners from 195 countries. We classified these countries according to the World Bank income categories for 2021, as high-income countries (59 countries, 30%), upper-middle income (58 countries, 30%), lower-middle income (47 countries, 24%), and low-income (31 countries, 16%) (https://datahelpdesk.worldbank.org/knowledgebase/articles/906519-world-bank-country-and-lending-groups). In order to inform our analyses we defined high-income countries as “Northern”, and countries in all other income categories as “Southern”. As a focus of our study was on the relationships between partners from different countries or within a country, we only included in the analyses those 2876 partnerships that specifically identified partners from at least one country. The remaining 1645 partnerships (36% of all recorded on the platform) only referred to an organisation (e.g. the UN or the European Commission) or a broader region, with most referring to “oceans” (863, 19%) or “global” (371, 8%).

Using the data in our excel database, we calculated and mapped the spatial distribution of the number and percentage of partners’ countries in different regions involved in the selected 2876 partnerships. We used a GIS world shape file downloaded from http://tapiquen-sig.jimdo.com (Carlos Efraín Porto Tapiquén. Orogénesis Soluciones Geográficas. Porlamar, Venezuela 2015. Based on shapes from Environmental Systems Research Institute, ESRI. Free Distribution) and the World Bank’s regions for this map and all subsequent mapping. We then calculated and mapped for each Northern and Southern country the number of partnerships that specifically identified partners from: (a) one or more countries; (b) only one country, i.e. domestic partnerships; (c) 2 to 20 countries; and (d) more than 20 countries. In doing so, we also calculated the average number of partnerships per partners’ countries in relation to the size of partnerships and country income category. We then identified each partnership’s focal SDGs and presented them in relation to the size of partnerships and the income category of partners’ countries.

We calculated the number of country-wise relationships within those partnerships that included partners from at least two countries. Outlying partnerships that involved partners from an exceptional number of countries (those more than 1.5 times the interquartile range above the third quartile) were excluded. This resulted in a dataset of 264 partnerships that included partners from between two and 20 countries. In order to analyse the country-wise relationships between partners, we assumed that a partner from a country had relations with partners from all other countries within the same partnership. For example, the largest partnership involving 20 countries had 190 country-wise relationships; Eq. (1):

$$\left(\genfrac{}{}{0pt}{}{20}{2}\right)=\frac{20!}{\left(20-2\right)! 2!}=190$$

However, we acknowledge that within each partnership some partners will be more involved than others and have a greater proportion of meaningful country-wise relationships. We then used these calculations to map the total number of country-wise relationships within and between different World Bank regions by type (i.e. North–North, North–South and South-South). The calculations also allowed us to determine the average number of country-wise relationships for Northern countries or Southern countries in different World Bank regions, separated into relationships between partners in different Northern or Southern countries, within or outside each region. Finally, we identified the average number of country-wise relationships for Northern or Southern countries in different World Bank regions by SDG.

All calculations and statistical analyses were conducted in the scientific programming and numeric computing platform MATLAB, while maps were produced using the geographic information system applications ARCGIS and QGIS. Figures were visually adjusted using the raster graphic editor GIMP and the vector graphic editor INKSCAPE.

## Supplementary Information

Supplementary Information.

## Data Availability

The raw data analysed in this paper are freely available from the UN’s SDG Partnerships Platform (https://sustainabledevelopment.un.org/partnership/browse).
