# Supplementary material for: SDG partnerships may perpetuate the global North–South divide
Source: Sci Rep. 2021 Nov 25;11:22092. doi: 10.1038/s41598-021-01534-6 (PMC8617181; doi:10.1038/s41598-021-01534-6)
Supplement: Supplementary file 1 — Supplementary Information. [file 41598_2021_1534_MOESM1_ESM.pdf]

SDG partnerships may perpetuate the global North-South divide

Malgorzata Blicharska, Claudia Teutschbein, Richard J. Smithers

Supplementary Material

Figure S1. Larger version of Figure 2. Distribution of the number of partnerships that specifically identified partners' countries. Overview of the number of partnerships that specifically identified partners from: (a) one or more countries; (b) only one country, i.e. domestic partnerships; (c) 2 to 20 countries; and (d) more than 20 countries. Northern countries (high income) are shown in shades of blue; Southern countries (upper-middle, lower-middle and low income) are shown in shades of red. Maps generated in QGIS 3.12 (https://qgis.org/).

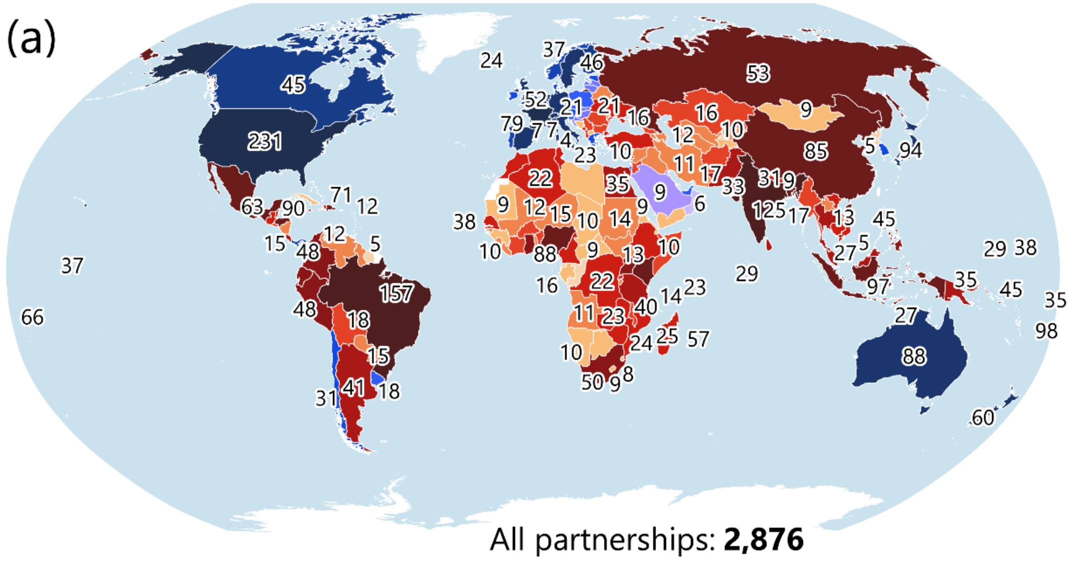

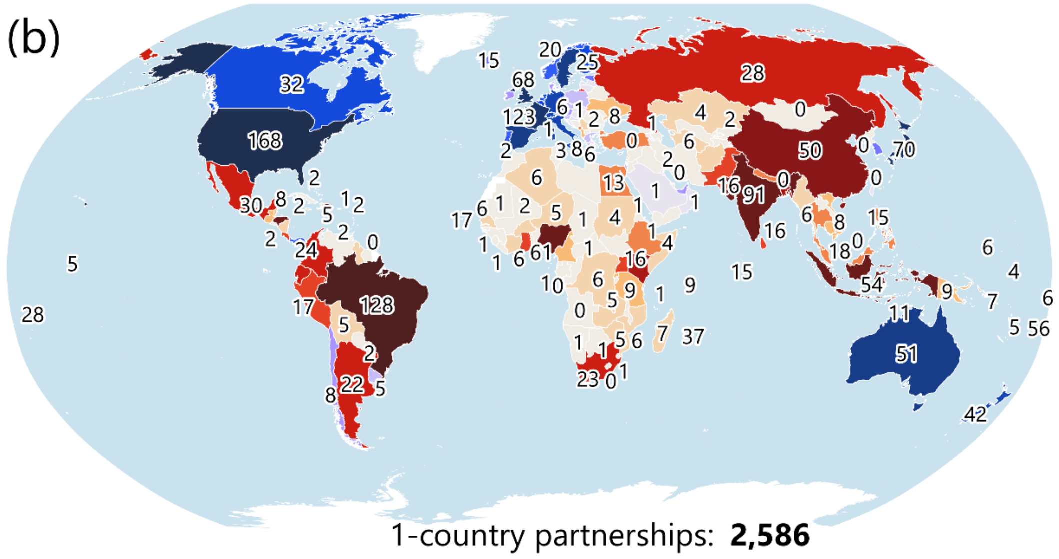

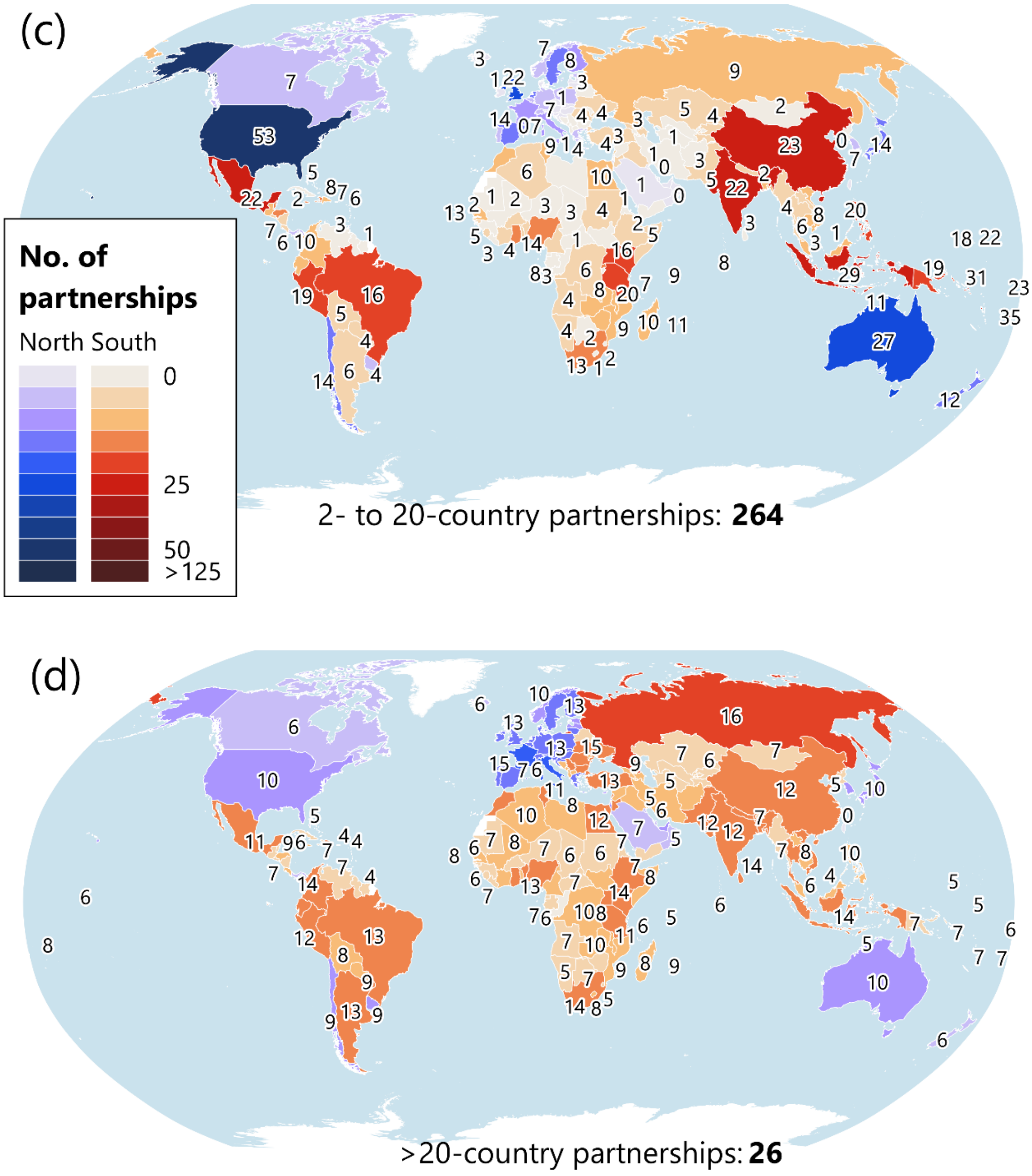

(c)
No. of partnerships
North South
0
25
50
>125
2- to 20-country partnerships: **264**
(d)
>20-country partnerships: **26**

Figure S2. Larger version of figure 4. Partnerships' focal SDGs. The SDGs addressed by (a) all partnerships that specifically identified partners from at least one country, (b) domestic partnerships, (c) partnerships involving 2 to 20 countries, and (d) partnerships with more than 20 countries. The bars on the left of Figures 4a to 4d represent the percentage of partnerships that address each SDG. The bars on the right of Figures 4a to 4d represent the percentage of partnerships that address each SDG involving partners from countries in each income category (represented by different intensities of colour). Note that many partnerships include partners from countries across multiple income categories and address more than one SDG.

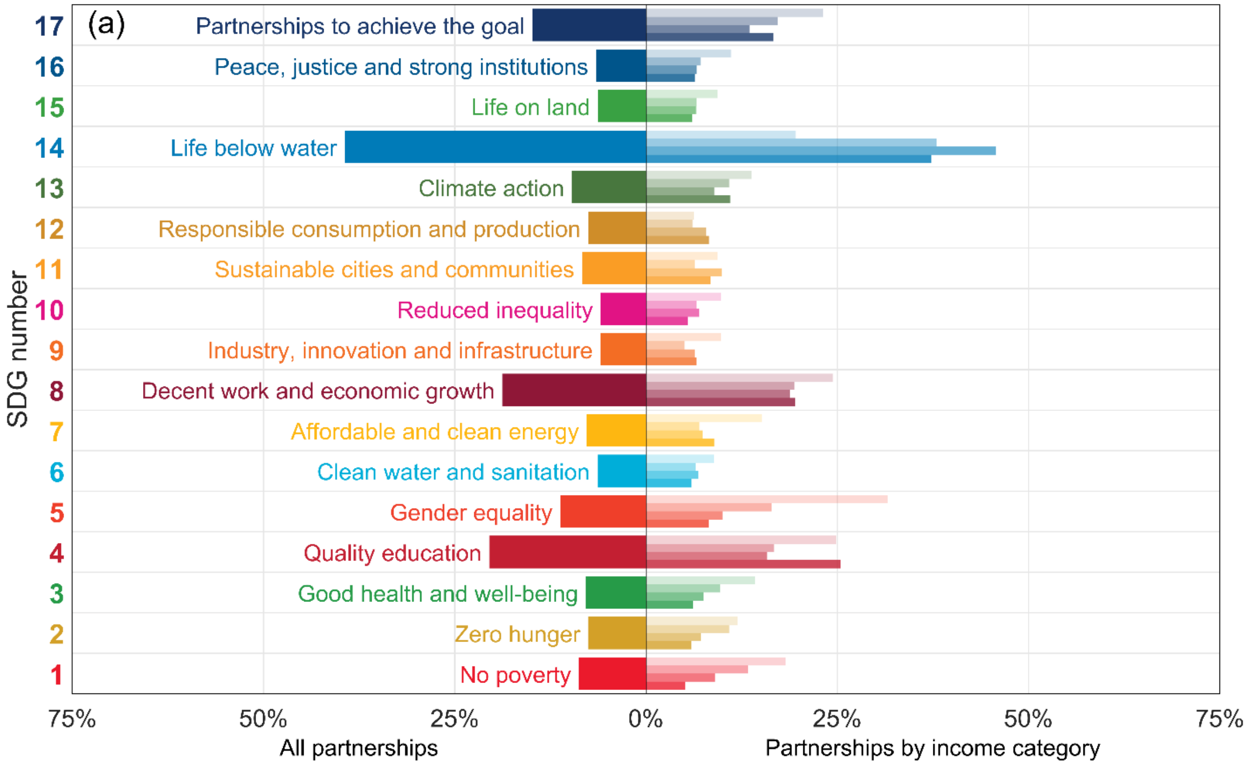

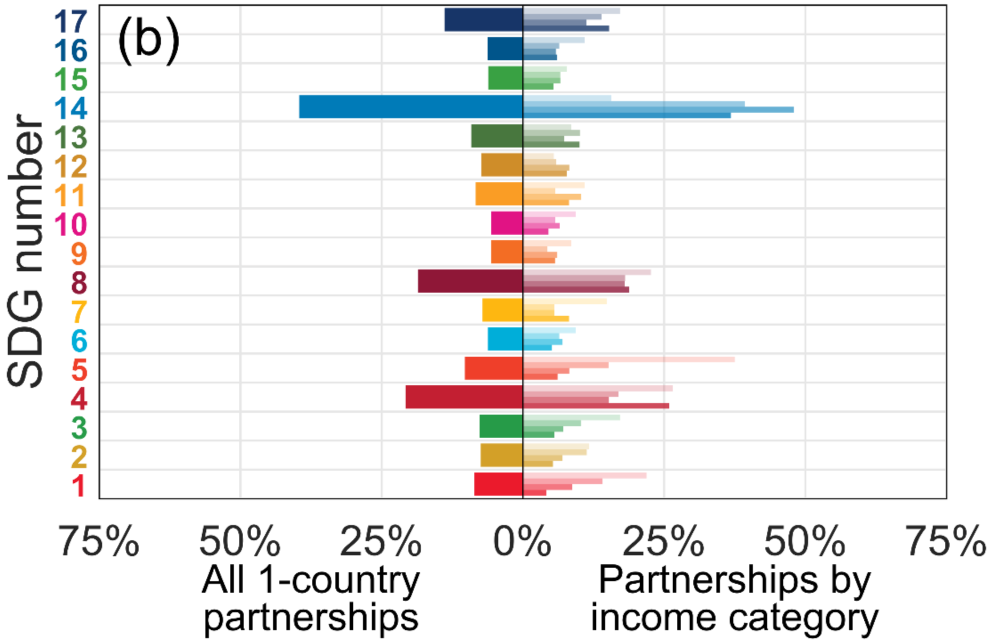

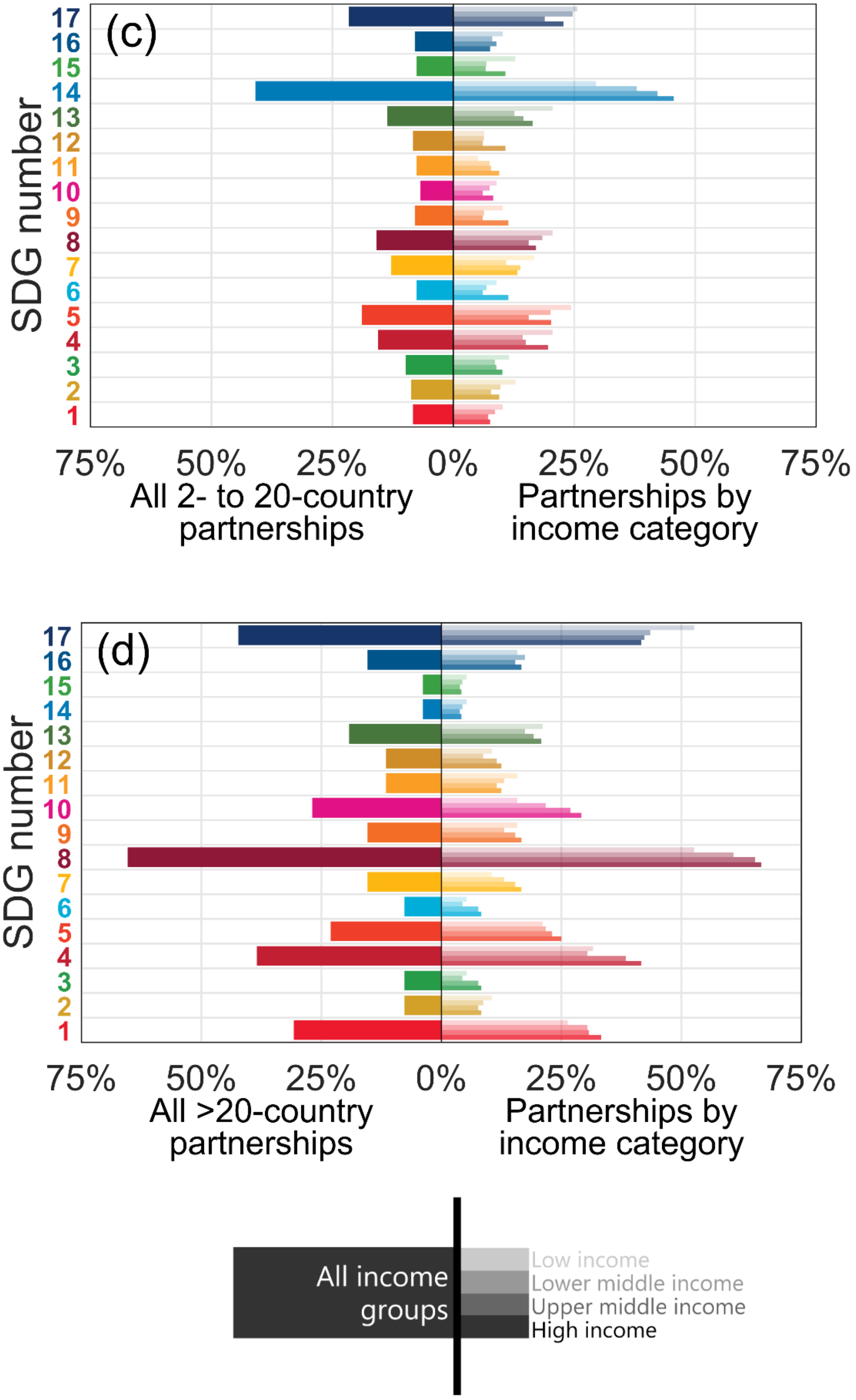
(c)
SDG number
17
16
15
14
13
12
11
10
9
8
7
6
5
4
3
2
1
75%
50%
25%
0%
25%
50%
75%
All 2- to 20-country partnerships
Partnerships by income category
(d)
SDG number
75%
50%
25%
0%
25%
50%
75%
All >20-country partnerships
Partnerships by income category
All income groups
Low income
Lower middle income
Upper middle income
High income

Figure S3. Larger version of Figure 5. Number of country-wise relationships within and between World Bank regions. The types of relationships are indicated by different coloured circles (within regions) and lines (between regions): (a) North-North relationships (black), (b) North-South relationships (grey) and (c) South-South relationships (white). The size of circles and the figures in the circles represent the number of relationships between partners from different countries within each region. Line thickness and the figures in italics associated with the lines represent the number of relationships between partners from different regions. Numbers in the coloured boxes of the legend identify the respective numbers of Northern countries and Southern countries in each region included in the analysis. Maps generated in Arcgis Desktop 10.7 (https://www.esri.com/en-us/arcgis/products/arcgis-desktop/overview) and further modified in Inkscape 0.92.5 (https://inkscape.org/).

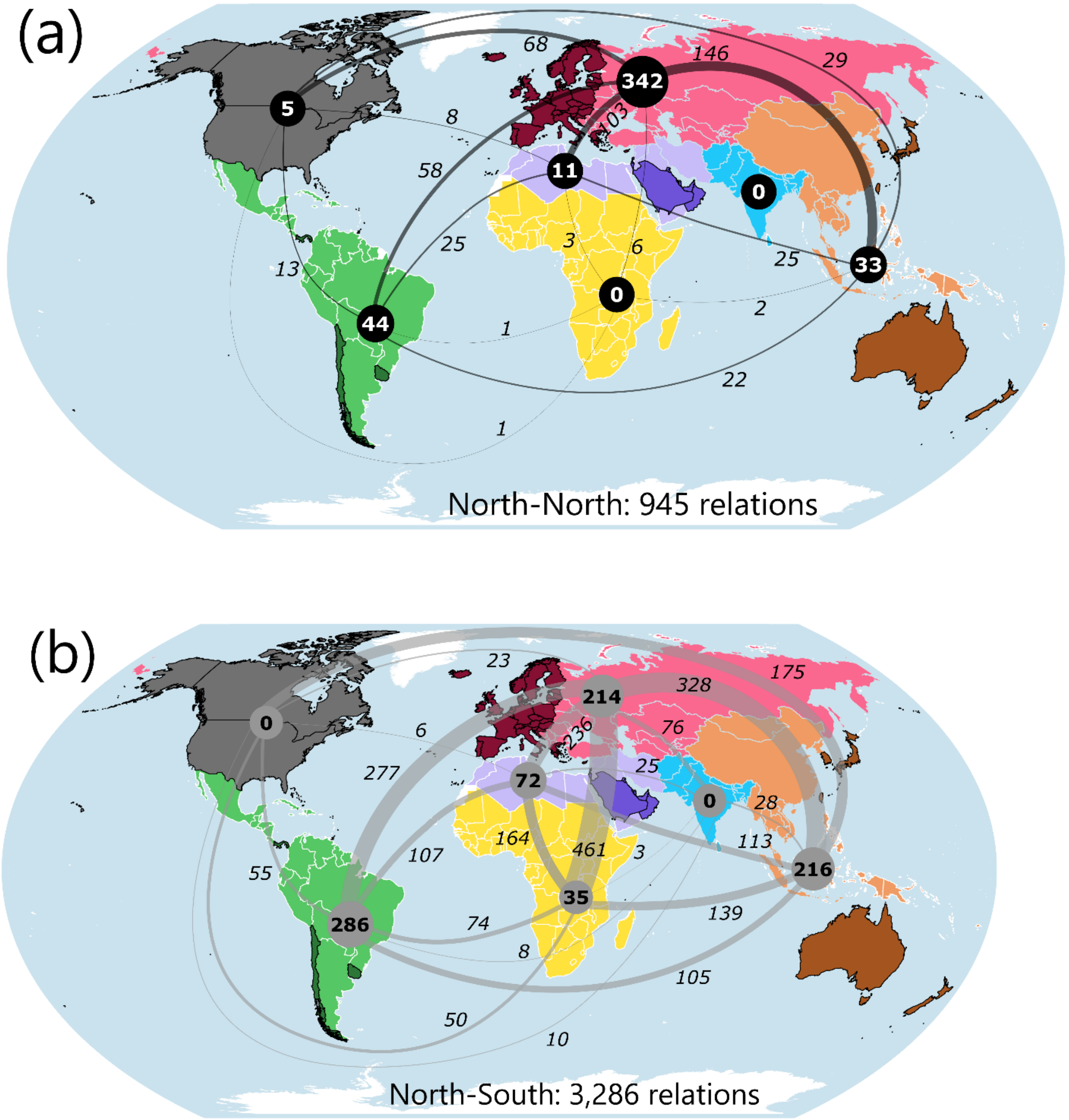

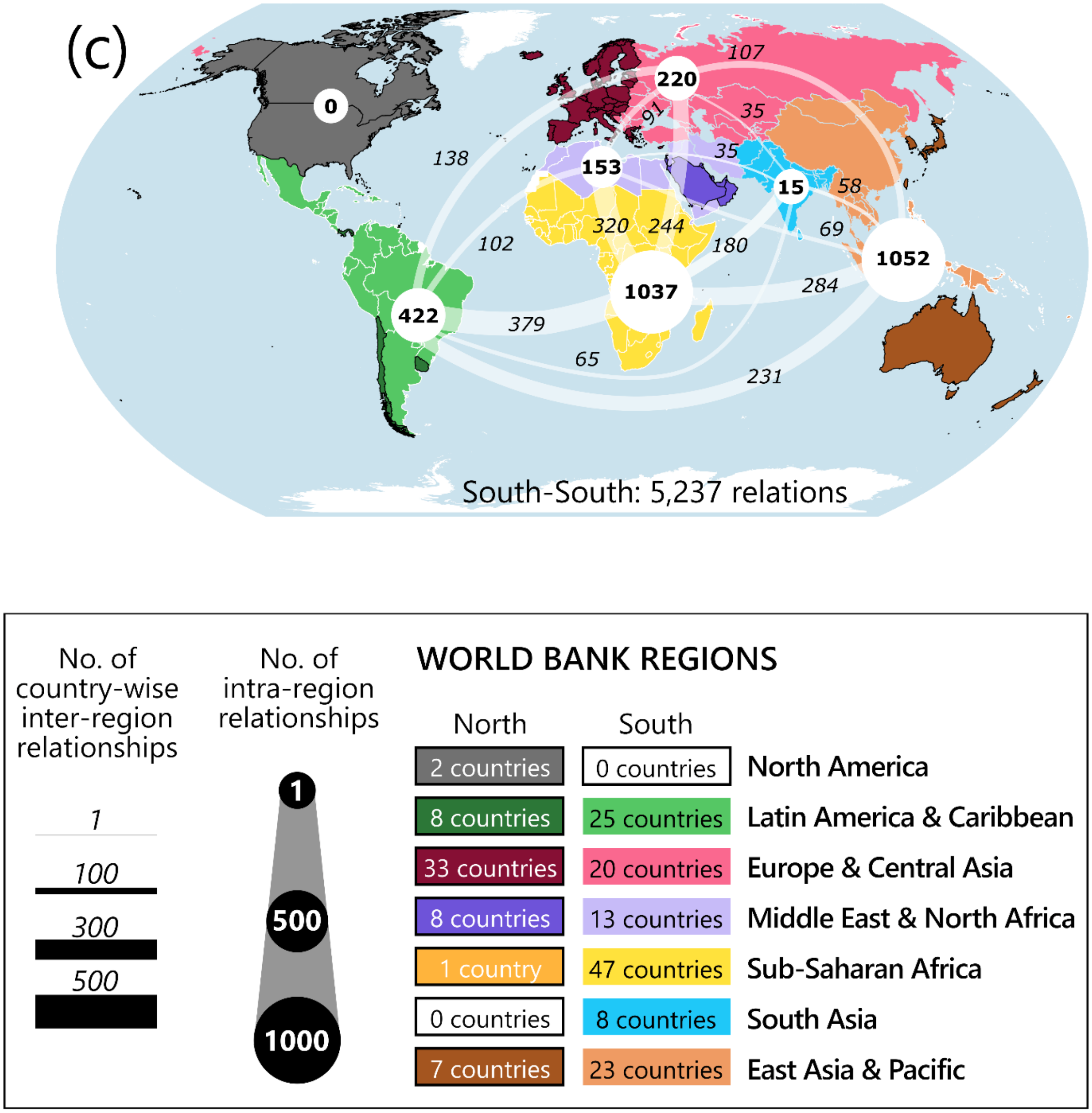
(c)
0
220
107
35
91
35
153
15
58
138
102
320
244
180
69
1052
1037
422
284
379
65
231
South-South: 5,237 relations
No. of country-wise inter-region relationships
1
100
300
500
No. of intra-region relationships
1
500
1000
WORLD BANK REGIONS
North
South
2 countries
0 countries
North America
8 countries
25 countries
Latin America & Caribbean
33 countries
20 countries
Europe & Central Asia
8 countries
13 countries
Middle East & North Africa
1 country
47 countries
Sub-Saharan Africa
0 countries
8 countries
South Asia
7 countries
23 countries
East Asia & Pacific

Figure S4. Larger version of figure 6. Comparison of the average number of country-wise relationships. For: (a) Northern countries or Southern countries in different World Bank regions (blue or red circles), separated into relationships between partners in different Northern or Southern countries (blue or red bars), within or outside each region (transparent or filled bars), where the number at the end of each bar is the percentage of that type of relationship within that region; (b) Northern countries in different World Bank regions by SDG; and (c) Southern countries in different World Bank regions by SDG.

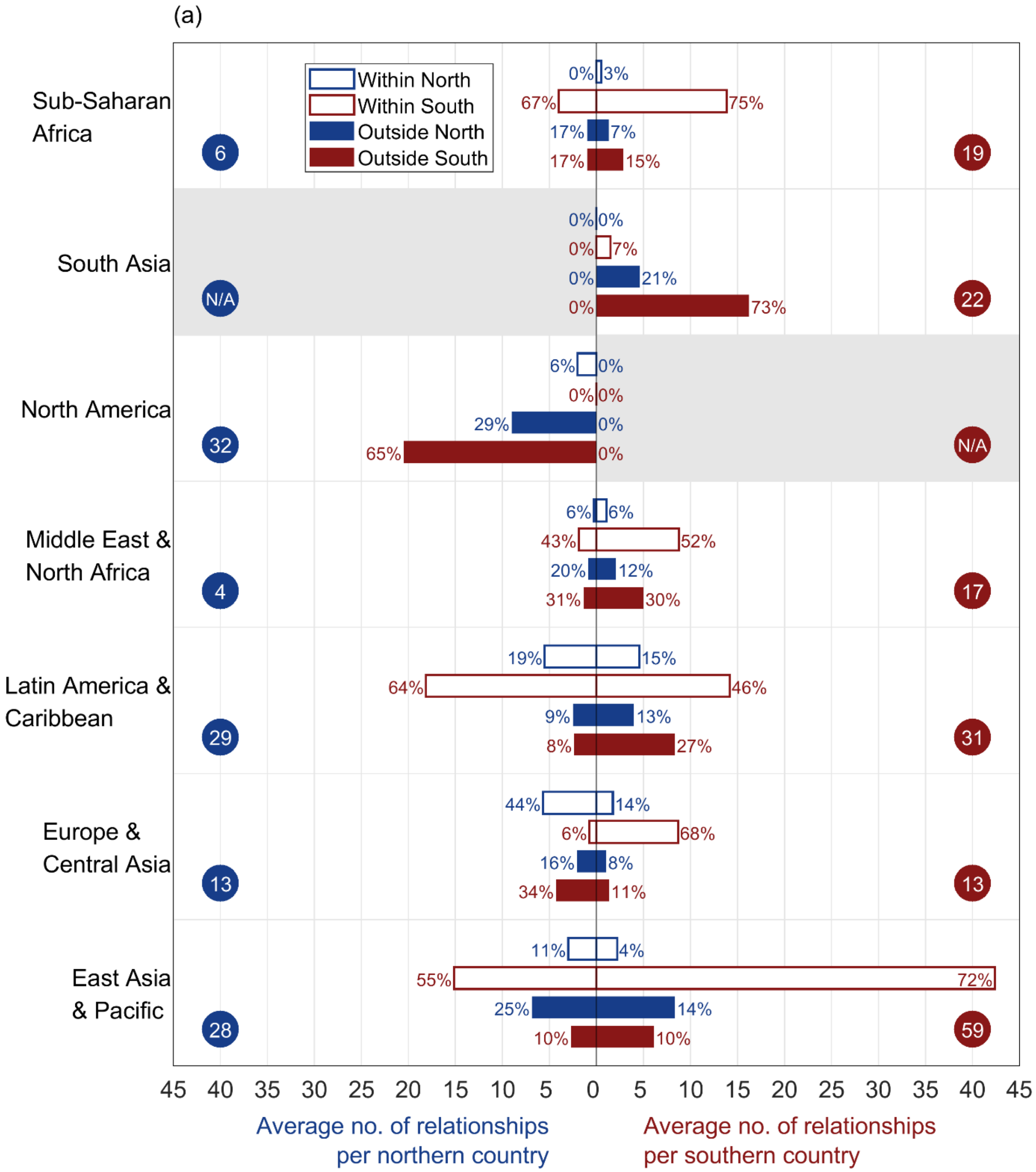

(c)

	1	2	3	4	5	6	7	8	9	10	11	12	13	14	15	16	17
Sub-Saharan Africa	2	5	1	5	5	1	2	9	0	2	1	0	3	4	1	1	5
South Asia	1	2	1	4	8	1	6	2	1	1	3	0	2	6	0	2	4
North America	-	-	-	-	-	-	-	-	-	-	-	-	-	-	-	-	-
Middle East & North Africa	4	9	3	11	12	6	3	15	4	7	3	3	8	8	7	4	7
Latin America & Caribbean	2	1	0	3	1	0	7	7	1	1	2	0	9	18	0	1	7
Europe & Central Asia	0	0	0	8	0	0	0	8	0	1	0	0	0	0	1	0	1
East Asia & Pacific	5	4	7	7	9	3	7	5	1	1	3	3	11	36	3	1	11

SDG number

(b)

	1	2	3	4	5	6	7	8	9	10	11	12	13	14	15	16	17
Sub-Saharan Africa	0	0	0	0	0	0	0	0	0	0	0	0	0	5	0	0	1
South Asia	-	-	-	-	-	-	-	-	-	-	-	-	-	-	-	-	-
North America	4	2	3	5	6	5	5	3	0	3	4	2	3	30	1	3	7
Middle East & North Africa	1	3	2	4	3	3	1	3	1	4	1	1	3	4	3	1	1
Latin America & Caribbean	1	0	0	2	2	1	11	4	0	1	1	0	8	19	0	1	4
Europe & Central Asia	0	2	0	13	2	2	1	3	0	11	2	0	2	7	2	0	3
East Asia & Pacific	3	0	1	4	6	1	1	5	0	1	3	1	0	12	0	3	5

SDG number
